# Organoid-Transplant Model Systems to Study the Effects of Obesity on the Pancreatic Carcinogenesis *in vivo*

**DOI:** 10.3389/fcell.2020.00308

**Published:** 2020-04-28

**Authors:** Francesca Lupo, Geny Piro, Lorena Torroni, Pietro Delfino, Rosalinda Trovato, Borislav Rusev, Alessandra Fiore, Dea Filippini, Francesco De Sanctis, Marcello Manfredi, Emilio Marengo, Rita Teresa Lawlor, Maurizio Martini, Giampaolo Tortora, Stefano Ugel, Vincenzo Corbo, Davide Melisi, Carmine Carbone

**Affiliations:** ^1^Section of Anatomical Pathology, Department of Diagnostic and Public Health, University of Verona, Verona, Italy; ^2^Medical Oncology, Department of Medical and Surgical Sciences, Fondazione Policlinico Universitario A. Gemelli IRCCS, Rome, Italy; ^3^Unit of Epidemiology and Medical Statistics, University of Verona, Verona, Italy; ^4^Section of Immunology, Department of Medicine, University of Verona, Verona, Italy; ^5^ARC-Net Research Centre, University of Verona, Verona, Italy; ^6^Department of Translational Medicine, Center for Translational Research on Autoimmune and Allergic Disease, University of Piemonte Orientale, Novara, Italy; ^7^Department of Sciences and Technological Innovation, University of Piemonte Orientale, Alessandria, Italy; ^8^Department of Translational Medicine and Surgery, Università Cattolica del Sacro Cuore, Rome, Italy; ^9^Section of Medical Oncology, Department of Oncology, University of Verona, Verona, Italy

**Keywords:** obesity, organoid models, pancreatic cancer, carcinogenesis, adipokines

## Abstract

Pancreatic ductal adenocarcinoma (PDAC) is the third leading cause of cancer-related mortality among adults in developed countries. The discovery of the most common genetic alterations as well as the development of organoid models of pancreatic cancer have provided insight into the fundamental pathways driving tumor progression from a normal cell to non-invasive precursor lesion and finally to widely metastatic disease, offering new opportunities for identifying the key driver of cancer evolution. Obesity is one of the most serious public health challenges of the 21st century. Several epidemiological studies have shown the positive association between obesity and cancer-related morbidity/mortality, as well as poorer prognosis and treatment outcome. Despite strong evidence indicates a link between obesity and cancer incidence, the molecular basis of the initiating events remains largely elusive. This is mainly due to the lack of an accurate and reliable model of pancreatic carcinogenesis that mimics human obesity-associated PDAC, making data interpretation difficult and often confusing. Here we propose a feasible and manageable organoid-based preclinical tool to study the effects of obesity on pancreatic carcinogenesis. Therefore, we tracked the effects of obesity on the natural evolution of PDAC in a genetically defined transplantable model of the syngeneic murine pancreatic preneoplastic lesion (mP) and tumor (mT) derived-organoids that recapitulates the progression of human disease from early preinvasive lesions to metastatic disease. Our results suggest that organoid-derived transplant in obese mice represents a suitable system to study early steps of pancreatic carcinogenesis and supports the hypothesis that inflammation induced by obesity stimulates tumor progression and metastatization during pancreatic carcinogenesis.

## Introduction

Cancer and obesity are the two major epidemics of the 21st century (Kaidar-Person et al., 2011). Pancreatic ductal adenocarcinoma (PDAC) is the third leading cause of cancer-related mortality among adults in the developed countries, with a median survival of few months and a 5-year survival of less than 6%. It is projected to become the second cause of cancer death in Western societies within a decade (Siegel et al., 2019). PDAC is associated with several distinct precursor lesions that likely impact disease biology, efficacy of therapy, and prognosis. These lesions include pancreatic intraepithelial neoplasia (PanIN), intraductal papillary mucinous neoplasm (IPMN), and mucinous cystic neoplasm (MCN) (Castellano-Megias et al., 2014). The stepwise progression of microscopic PanIN lesions to invasive PDAC has been well characterized.

Organotypic cultures are a manageable tool to identify and interrogate pathways involved in pancreatic carcinogenesis (Froeling et al., 2010). We recently developed the method to establish and characterize organoids from mouse models of spontaneous pancreatic cancer (Ponz-Sarvise et al., 2015; Filippini et al., 2019).

These genetically engineered mouse models of PDAC were obtained through the Cre-Lox technology and the conditional activation of mutant endogenous alleles of the Kras (KC) (Hingorani et al., 2003) and Kras and Trp53 genes (KPC) (Hingorani et al., 2005) under the expression of the pancreas-specific Pdx-1 promoter.

Indeed, preclinical evidence indicated that a “sensitizer” background (specifically oncogenic Kras activation) is a necessary prerequisite when trying to identify factors (genetic or non-genetic) that accelerate progression of pancreatic cancer in mouse models (Perez-Mancera et al., 2012) where genes’ inactivation due to sleeping beauty transpositions was not sufficient to drive tumor formation in the absence of Kras activation. This reflects on the nearly universal Kras mutation in PDAC and the well-established consensus that oncogenic mutation of Kras is the initiating event in pancreatic carcinogenesis. Therefore, our intent has been to specifically evaluate whether or not diet-induced obesity was able to cooperate with oncogenic Kras (G12D mutation) to accelerate tumorigenesis and to promote tumor progression in our model and, consequently, if the model itself could be used as a biological source for the identification of biomarkers and determinants of disease progression.

Moreover, solid epidemiological evidence connects obesity with incidence, stage, and survival in PDAC. However, the underlying mechanistic basis linking obesity to PDAC pathogenesis and development remain largely elusive and a realistic comprehensive model of obesity and pancreatic carcinogenesis is still under construction (Larsson et al., 2007; Yuan et al., 2013; Himbert et al., 2017). The C57BL6 mouse, under specific stimulation (diet or genetic alteration), could recapitulate human metabolic unbalance that is observed in obesity, resulting in a useful well-characterized model system. Indeed, mice fed *ad libitum* with a high-fat diet (HFD) developed obesity, hyperinsulinemia, hyperglycemia, and hypertension, whereas no metabolic abnormality was observed when fed *ad libitum* with chow diet (Collins et al., 2004; Wang and Liao, 2012).

The most compelling preclinical evidence indicates that a HFD can accelerate pancreatic neoplasia in the conditional K-Ras^G12D^ (PDX1-CRE) mouse model (Dawson et al., 2013). A cross-talk between adipocytes, tumor-associated neutrophils, and pancreatic stellate cells has been described to promote desmoplasia, accelerate growth and impair delivery/efficacy of chemotherapeutics in models of established pancreatic cancer, with IL1β secreted by all these cells playing a major role in this cooperation (Incio et al., 2016). Peri-tumor adipocytes predict poor prognosis in multiple cancers (Hasebe et al., 2000; Yamaguchi et al., 2008), and promote proliferation and invasion of multiple types of cancer cells in *in vitro* and *in vivo* models (Tokuda et al., 2003; Zhang et al., 2009; Dirat et al., 2011; Nieman et al., 2011). Similar data support the role of steatosis in human tendency to PanIN, PDAC, and to more advanced disease (Mathur et al., 2009; Rebours et al., 2015), while human adipose tissue stem cells promote *in vitro* pancreatic cell proliferation and invasion (Ji et al., 2013). Finally, pancreatic adipocytes are associated with PDAC progression in murine models (Zyromski et al., 2009; Grippo et al., 2012; Meyer et al., 2016).

In a recent study, Sasaki et al. (2018) also showed that the reduction of apical extrusion was more evident when mice were fed an omega-6 fat diet such as soybean oil, compared to an omega-3 fat diet such as linseed oil. More importantly, in this study, data on higher inflammatory cytokines as well as macrophage infiltration in the first subgroup of mice, together with the evidence of an increased frequency of apical extrusion in HFD mice treated with aspirin, demonstrated a link between HFD and inflammation in pancreatic cancer. Indeed, inflammatory cytokines as well as macrophage infiltration, were higher in the first subgroup of mice, thus demonstrating a link between HFD and inflammation in pancreatic cancer, so far that aspirin treatment increased the frequency of apical extrusion in HFD mice (Sasaki et al., 2018).

We recently demonstrated that factors secreted by adipocytes induced epithelial-to-mesenchymal transition (EMT) and increased aggressiveness in two pancreatic cell transformation model system by orchestrating a complex paracrine signaling of soluble modulators of the non-canonical WNT signaling pathway that impinges upon activation and nuclear translocation of WNT receptor ROR2 (Carbone et al., 2018).

However, a comprehensive analysis aimed at directly identifying the paracrine molecular networks linking obesity to PDAC progression has not been performed yet.

In this study, we propose the use of organoid cultures generated from pancreas with different disease stages to study the effects of obesity on the pancreatic carcinogenesis. Therefore, we tracked the effects of obesity on the natural evolution of PDAC in a genetically defined transplantable model of syngeneic murine pancreatic preneoplastic (mP) and tumoural (mT) derived organoids that recapitulates the progression of the human disease from early preinvasive lesions to metastatic disease.

## Materials and Methods

### Organotypic Cultures

Detailed procedures to isolate preneoplastic and neoplastic pancreatic ducts have been described previously (Huch et al., 2013). Briefly, pre-neoplastic pancreatic ducts derived from *“Pdx1-Cre; Kras^+/LSL–G12D^* (KC)” (Hingorani et al., 2005), were manually picked after enzymatic digestion of the pancreas in DMEM medium containing 1% FBS (Gibco), 5 mg/ml Collagenase Type XI (Gibco) (digestion medium), 1 mg/mL Dispase II (Gibco); to establish primary tumor derived pancreatic organoids from tumor-bearing *Pdx1-Cre; Kras^+/LSL–G12D^; Trp53^+/LSL–R172H^* (KPC) mice (Hingorani et al., 2005), bulk tumor tissue was minced and digested in digestion medium for 2 h at 37°C with gentle rocking. Isolated material was incubated with TrypLE (Gibco) at 37°C for 10 min, embedded into growth factor-reduced Matrigel (Corning), and cultured in mouse complete medium [AdDMEM/F12 (Gibco) supplemented with 1% penicillin/streptomycin (Gibco), 1% GlutaMAX (Gibco), 10 mM HEPES (Gibco), B27 supplement (1X final) (Gibco), 1.25 mM N-Acetylcysteine (Sigma), 10% (v/v) Rspo1-conditioned media, 10 mM Nicotinamide (Sigma), 10 nM recombinant human-gastrin I (Tocris), 50 ng/ml recombinant mouse EGF (Gibco), 100 ng/ml recombinant human FGF10 (Peprotech), 0.5 μM A83-01 (Tocris), and 100 ng/ml recombinant human Noggin (Peprotech)].

To generate pancreatic tumor progression models, organoids were harvested from Matrigel using ice-cold Cell Recovery Solution (Corning) for 60 min, and then mechanically dissociated through fire-polished glass Pasteur pipettes. Before orthotopic transplantation, organoids (1 × 10^6^ cells/mouse) were resuspended in 50 μl/mice of a 2:3 dilution of Matrigel and cold 1X PBS (Gibco) (Boj et al., 2015).

### Obese Mice Models

Male C57BL/6 WT and leptin-deficient mice (C57BL/6J; ob/ob) were obtained from Charles River. Mice were at the age of 4 weeks divided in three groups fed with different chow (Brogaarden, Lynge, Denmark). A group receiving standard rodent chow, a group receiving a HFD with 60% of calories from fat, and a group receiving Low-Fat Diet (LFD) for 10 weeks (*n* = 20). All mice were housed and treated in accordance with the guidelines of the University of Verona Animal Ethic Committee.

### High-Resolution Ultrasound Imaging Acquisition and Analysis

Mice were anesthetized with 1.5–2% isoflurane in oxygen and restrained on a heated stage during imaging. Prewarmed ultrasound coupling gel (Aquasonic 100, Parker Laboratories, Inc., Fairfield, NJ, United States) was applied to the depilated skin before the imaging. Ultrasound imaging scan was performed using VisualSonics Vevo 2100 Ultrasound (VisualSonics Inc., Toronto, ON, Canada). 2D images of pancreas and neighbor anatomies were acquired using a linear-array transducer (MS-400) in B-mode with a 30-MHz center frequency that produces a 15-mm × 15-mm field of view at the 12-mm focal depth. The pancreas was manually delineated in parallel slices in the 3D serials. The areas of the outlined contours were summed and multiplied by the interslice distance to compute tumor volume. Exponential volume growth curves were fit by linear regression; coefficients of determination were computed to assess the goodness of fit of the exponential function using a GraphPad Prism 6 package (GraphPad, La Jolla, CA, United States).

### Histology

Tissues were fixed in 10% neutral buffered formalin and embedded in paraffin. Sections were subjected to H&E, Alcian Blue, Nuclear Fast Red, and Masson’s Trichrome staining as well as immunohistochemical staining. A pathologist who was blinded to all mice and organotypic information reviewed the H&E stained slides of the pancreatic tissue. The total number of PanIN lesions of different histologic grades (from low-grade to invasive carcinoma) in all pancreatic tissue slides of each case was counted according to Basturk et al. (2015).

When PanIN lesions of different grades were present in the same pancreatic duct, they were counted as separate PanIN lesions. Abrupt transition of highly atypical epithelial cells to the normal ductal epithelium was considered as cancerization of the ducts. The score was from precursor lesion of ducts to invasive carcinoma.

The following primary antibodies were used for immunohistochemical staining and established procedures: CD8 (14-0808-82, Thermo Scientific) 1:2000; Ly6G (ab25377 AbCam) 1:2000, CD11b (ab133357 AbCam) 1:4000; KI67 (9129 Cell Signaling) 1:4000.

### Multiplex Cytokines Profiling

Using Luminex XMAP multi-plexing technology (Bioplex 200, Bio-Rad s.r.l.), all plasma specimens were analyzed for TNFα, MIP1α, MIP1-β, MCP1, IL1b, IL-6, IL10, IL17, KC, and G-CSF. All Luminex assays were performed according to the instructions provided by the manufacturer (Bio-Rad Laboratories). Median fluorescence intensities were collected on a Luminex-200 instrument, using Bio-Plex Manager software version 6.2. Standard curves for each cytokine were generated using the premixed lyophilized standards provided in the kits. Cytokines concentrations in samples were determined from the standard curve using a 5-point regression.

### Flow Cytometry Analysis of Peripheral Blood and Tumor-Infiltrating Immune Cells

Circulating and tumor-infiltrating cells components were stained with antibody staining panels: a PBMC subset panel and a T cell subset panel. The PBMC subset panel antibody cocktail (CD3-PE CF594, CD4-FITC, CD8-PerCP Cy5.5, CD11b-AlexaFluor 700, Ly6G-APC, CD19-PE-Cy5, CD45-AmCyan, F4/80-FITC, DC-PE) was used to stain 100 μL whole blood in BD Trucount Tubes to determine numbers of various peripheral immune cell types.

### RNA-Sequencing (RNAseq)

Total RNA was isolated from pancreata tissue samples using the Trizol reagent (Invitrogen), following the manufacturer’s instructions. RNA was quantified using the NanoDrop (Thermo Scientific^TM^ NanoDrop 2000) and purity of samples was checked on 1% agarose gels for evaluating the 28S and 18S ribosomal RNA bands (28S/18S ratio). All samples with a ratio (28S/18S) of above 1.8 and an OD 260/280 ratio greater than 1.9 were sent to BGI Company in China for sequencing. RNA integrity number (RIN) was also measured on an Agilent Bio Analyzer 2100 system. Only RNA samples with a RIN > 7 were used for cDNA library construction. All cDNA libraries were sequenced using paired-end strategy (read length 150 bp) on an Illumina HiSeq 2000 platform. The raw RNA-Seq data were deposited and released in GEO Database (NCBI^1^).

Quality of raw reads was checked with FASTQC (Leggett et al., 2013). Transcripts were quantified with the alignment-free method implemented in Salmon 0.11.3 (Patro et al., 2017). Mouse genome and transcriptome from Gencode Release M18 (GRCm38.p6) were used. Quantified transcripts were imported to the statistical software R with the tximport package (Soneson 2015) and aggregated to the gene level using the option tx2gene. The matrix of gene counts was then converted to a DESeq data set with the function DESeqDataSetFromTximport function implemented in DEseq2 package version 1.22.2 (Love et al., 2014) and normalized using the rlog function. Differentially expressed genes were identified with the same package. Gene set variation analysis was performed on log2 normalized counts with the GSVA package 1.30.0 (Hanzelmann et al., 2013) using custom and MSigDB gene sets. Gene set enrichment analysis was performed with the fgsea package applying 10,000 permutations, Benjamini–Hochberg procedure for correcting *p*-values, and a *p*-value cut-off of 0.05. Heatmaps were generated either with the Bioconductor packages ComplexHeatmaps 1.20.0 or heatmap.

### Proteomic Analysis

#### Sera Sample Preparation

Twelve microliters of mouse sera were depleted of high abundant proteins using the Seppro Mouse spin column kit (Sigma-Aldrich Inc., St. Louis, MO, United States) following the manufacturer protocol. The method is used to bind mice Serum Albumin, IgG, Fibrinogen, Transferrin, IgM, Haptoglobin and alpha1-Antitrypsin and thus to increase the identification of low-abundant proteins. The sample was transferred into an Amicon Ultra-0.5 mL 3 kDa centrifugal filter (Millipore, Billerica, MA, United States) to collect the high molecular weight proteins. The sample was then subjected to denaturation with TFE, to reduction with DTT 200 mM, alkylation with IAM 200 mM and the complete protein trypsin digestion with 2 μg of Trypsin/Lys-C (Promega, Madison, WI, United States). The peptide digests were desalted on the Discovery < ^®^ DSC-18 solid phase extraction (SPE) 96-well Plate (25 mg/well) (Sigma-Aldrich Inc., St. Louis, MO, United States). After the desalting, the sample was vacuum evaporated and reconstituted with 20 μL of 0.05% formic acid in water.

#### LC-MS/MS Analyses

The sera proteins were analyzed with a micro-LC Eksigent Technologies (Eksigent Technologies, Dublin, CA, United States) system that included a micro LC200 Eksigent pump with flow module 5–50 μL, interfaced with a 5600+ TripleTOF system (Sciex, Concord, ON, Canada) equipped with DuoSpray Ion Source and CDS (Calibrant Delivery System). The stationary phase was a Halo C18 column (0.5 × 100 mm, 2.7 μm; Eksigent Technologies, Dublin, CA, United States). The mobile phase was a mixture of 0.1% (v/v) formic acid in water (A) and 0.1% (v/v) formic acid in acetonitrile (B), eluting at a flow-rate of 15.0 μL min^–1^ at an increasing concentration of solvent B from 2% to 40% in 30 min. The injection volume was 4.0 μL and the oven temperature was set at 40°C. For identification purposes the samples were subjected to a data dependent acquisition (DDA): the mass spectrometer analysis was performed using a mass range of 100–1500 Da (TOF scan with an accumulation time of 0.25 s), followed by a MS/MS product ion scan from 200 to 1250 Da (accumulation time of 5.0 ms) with the abundance threshold set at 30 cps (35 candidate ions can be monitored during every cycle). The ion source parameters in electrospray positive mode were set as follows: curtain gas (N2) at 25 psig, nebulizer gas GAS1 at 25 psig, and GAS2 at 20 psig, ionspray floating voltage (ISFV) at 5000 V, source temperature at 450°C and declustering potential at 25 V.

For the label-free quantification, the samples were subjected to cyclic data independent analysis (DIA) of the mass spectra, using a 25- Da window: the mass spectrometer was operated such that a 50-ms survey scan (TOF-MS) was performed and subsequent MS/MS experiments were performed on all precursors. These MS/MS experiments were performed in a cyclic manner using an accumulation time of 40 ms per 25-Da swath (36 swaths in total) for a total cycle time of 1.5408 s. The ions were fragmented for each MS/MS experiment in the collision cell using the rolling collision energy. The MS data were acquired with Analyst TF 1.7 (Sciex, Concord, ON, Canada). Two DDA and three DIA acquisitions were performed.

##### Protein Database Search

The DDA files were searched using Protein Pilot software v. 4.2 (Sciex, Concord, ON, Canada) and Mascot v. 2.4 (Matrix Science Inc., Boston, MA, United States). Trypsin as digestion enzyme was specified for both the software. For Mascot we used 2 missed cleavages, the instrument was set to ESI-QUAD-TOF and the following modifications were specified for the search: carbamidomethyl cysteine as fixed modification and oxidized methionine as variable modification. A search tolerance of 50 ppm was specified for the peptide mass tolerance, and 0.1 Da for the MS/MS tolerance. The charges of the peptides to search for were set to 2+, 3+, and 4+, and the search was set on monoisotopic mass.

The UniProt Swiss-Prot reviewed database containing mouse proteins (version 20july15, containing 23,304 sequence entries) was used and a target-decoy database search was performed. False Discovery Rate was fixed at 1%.

##### Protein Quantification

The quantification was performed by integrating the extracted ion chromatogram of all the unique ions for a given peptide. SwathXtend was employed to build an integrated assay library, built with the DDA acquisitions, using a protein FDR threshold of 1%. The quantification was carried out with PeakView 2.0 and MarkerView 1.2 (Sciex, Concord, ON, Canada). Six peptides per protein and six transitions per peptide were extracted from the SWATH files. Shared peptides were excluded as well as peptides with modifications. Peptides with FDR lower than 1.0% were exported in MarkerView for the *t*-test.

### Overall Survival Analysis

Survival analyses were performed using the computing environment R and the packages survival, for fitting the model, and survminer for plotting. Overall survival data were obtained from the link https://dcc.icgc.org/releases/current/Projects/PACA-AU. Median survival was estimated with the Kaplan-Meier method and the difference was tested using the log-rank (Mantel-Cox) test. For stratifying survival, gene expression data were divided into quartiles.

## Results

### *In vivo* Modeling of Organoid Transplant Systems Recapitulates Human Pancreatic Carcinogenesis

To generate diet-induced obesity (DIO), C57BL6/J mice were fed a LFD (*n* = 20) or HFD (*n* = 20). In addition, we used a genetic-induced-obesity (GIO) model of leptin deficiency (ob/ob) C57BL6/J mice fed with normal chow (*n* = 10). Mice were weighed and flanks measured weekly (Supplementary Figures 1A,B). After 10 weeks, the HFD mice reached the obesity rate (e.g., 1,5 the weight of lean mice), measured as gain in weight and flank length (Supplementary Figures 1C,D).

Using established procedure (Boj et al., 2015), we generated the mP and mT organoid cultures from the pancreatic tissue of KC (Kras^+/LSL–G12D^; PDX-1-Cre) and KPC (Kras^+/LSL–G12D^; Trp53^+/LSL–R172H^; Pdx1-Cre) (Hingorani et al., 2005) mice (*n* = 3), respectively.

We initially established the two organoid-transplant model systems, by orthotopically injecting preinvasive preneoplastic mP and neoplastic mT pancreatic organoid cultures in DIO and GIO models. Briefly, each mP and mT pancreatic organoid model was injected orthotopically in HFD (*n* = 10), LFD (*n* = 10), and ob/ob mice (*n* = 5). We and other groups have previously demonstrated that orthotopic transplants of mouse tumor organoids in syngeneic immunocompetent mice slowly progress from preinvasive lesions (PanIN-like lesions) to invasive carcinomas. The neoplastic progression was associated with the expansion of myeloid cells, particularly of granulocytes, and accumulation of M2 macrophages, which inversely correlated with CD8+ cell infiltration (Filippini et al., 2019).

Two weeks after orthotopic organoids injections mice were analyzed by high-resolution ultrasound capture system to monitor the engraftment incidence rate as the presence of detectable mass at the pancreas and the growth of mP and mT organoid–derived cells.

**FIGURE 1:**
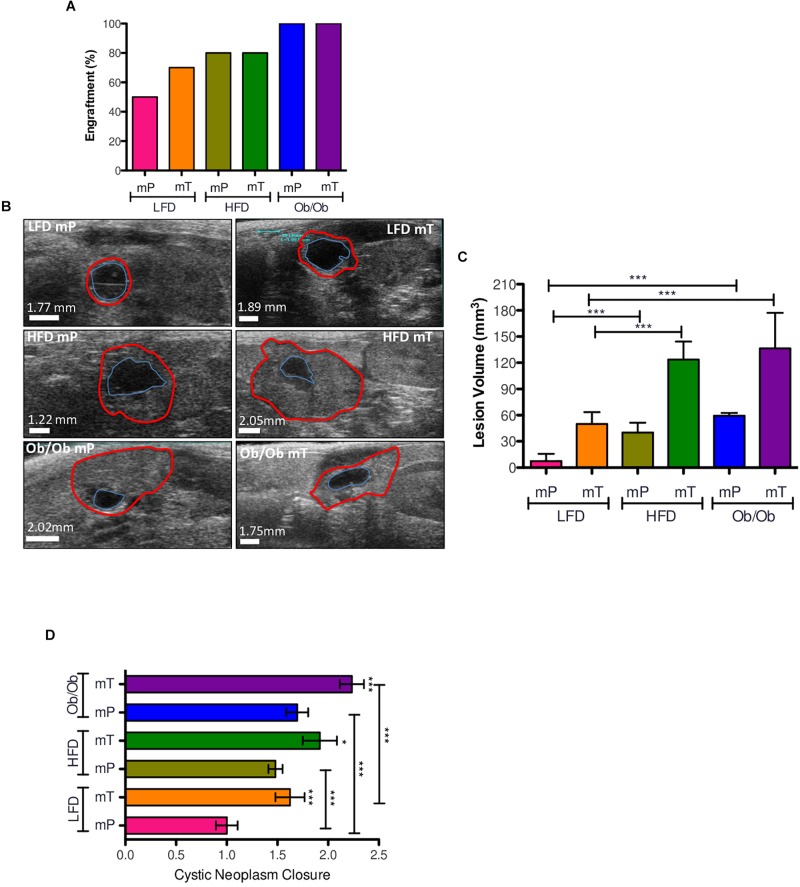
Effect of obesity on organoid-transplant models engraftment and growth. **(A)** Obesity accelerated the engraftment rate of preinvasive preneoplastic (mP) and neoplastic (mT) organoid models. Engraftment rate was represented as percentage of mice with pancreatic cysts respect to all mice of each group. **(B)** Ultrasound imaging of pancreatic cysts; the volume was reports as the increase of external epithelium (red line). **(C)** Volume analysis of pancreatic cysts. Data of each group were reported as mean and 95%CI; **(D)** cystic neoplasm closure. All the measures were taken with ultrasound technology (VEVO 2100). *, *p* < 0.05; ***, *p* < 0.001; by two-tailed unpaired Student’s *t*-tests. The borders distance of each cyst was measured with ImageJ software; Red line, external epithelium; Blue line, internal epithelium.

As expected, in lean mice the engraftment rate of mT was higher compared to mP models according to the more aggressive behavior of mT organoids. Ultrasound imaging analysis showed that obesity positively increased the engraftment rate of both mP and mT organoids–derived cells (Figure 1A), and in particular was able to increase the engraftment rate of the preinvasive mP organoid model.

The natural evolution of *in vivo* transplanted organoid model was from a small solid lesion to an invasive carcinoma through a cystic structure recognizable as a spherical black region with distinct borders. To demonstrate that obesity affected this model of evolution we analyzed the growth and progression of our mP and mT organoid models in both DIO and GIO mice models by ultrasound imaging system.

As expected, obesity significantly promoted the growth of both precancerous mP and cancerous mT organoid models (Figures 1B,C and Supplementary Figure 2A) with an increase of cystic neoplasms closure in comparison to lean mice (Figure 1D).

These data suggested that obesity increased the engraftment rate and growth of both mP and mT organoid models of pancreatic carcinogenesis.

To evaluate the effects of obesity on pancreatic carcinogenesis, we performed histological analysis of both mP and mT pancreatic-derived tissues.

**FIGURE 2:**
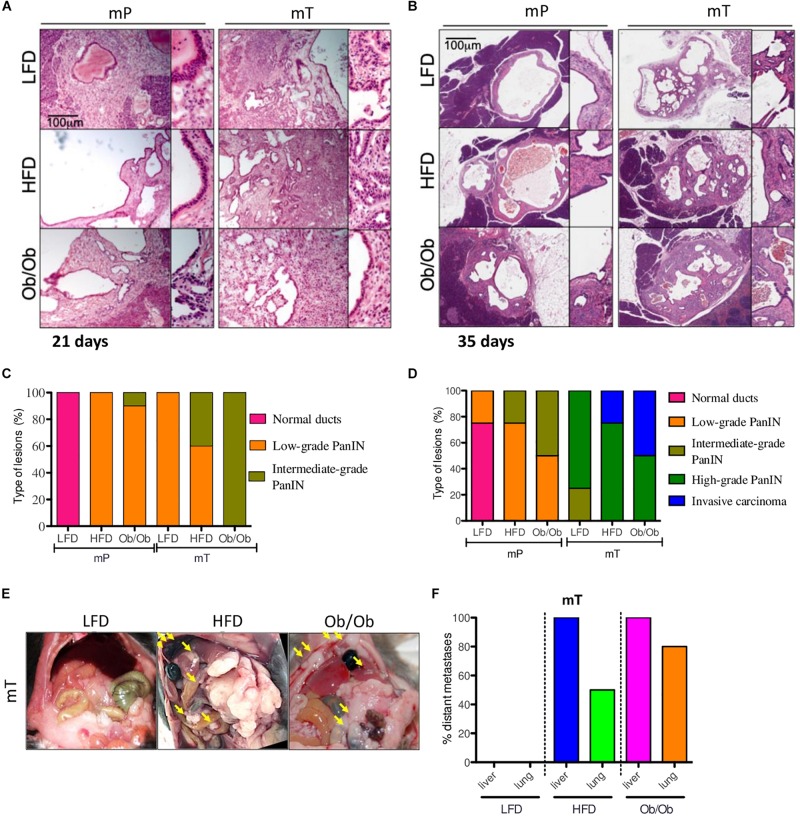
Obesity accelerated the carcinogenesis and growth of both mP and mT pancreatic organoid models. Representative micrographs of pancreatic cysts of ob/ob, HFD and LFD mice bearing mP and mT organoid models. Hematoxylin & eosin staining, original magnifications: 100x and 200x, scale bar 100 μm; The H&E stained slides of the pancreatic tissue excised **(A)** at 21 days and **(B)** 35 days. Each lesion was reviewed by a pathologist and a score of progression assigned (normal stratification of ducts; to invasive carcinoma **(C,D)**; **(E)** Representative images of peritoneal metastasis in the mT organoid bearing mice (groups: mT_LFD = 3; mT_HFD = 3; mT_ob/ob = 2); **(F)** Quantification of retro-peritoneal metastasis in the mT organoid bearing mice (groups: mT_LFD = 3; mT_HFD = 3; mT_ob/ob = 2). Data are shown as the percentage of mice with metastases in the indicated site. The picture reported the percentage of mice presenting with at least one metastasis in indicated organs.

In particular, time-course analysis of both mP and mT pancreatic-derived tissues revealed that both GIO and DIO promoted pancreatic cancer progression displaying a significant difference in time of progression. While at 21 days from organoid injection mP showed low or no dysplastic events in ductal epithelial cells, the mT model showed an increased dysplasia with several PanIN low-grade lesions in HFD and ob/ob mice compared to LFD mice (Figures 2A,B). At 35 days, histopathological analysis of obese mice showed an increase in cancer progression of both mP and mT lesions, measured as the difference in the number and quality of PanIN lesions compared to lean mice. This is particularly evident for GIO and DIO mT models that showed an increase of invasive carcinoma compared to the lean mice models (Figures 2C,D) as well as a regulation of two pancreatic carcinoigenesis MUC5AC and MUC6 (Supplementary Figure 3).

Similar to previous studies (Incio et al., 2016; Sasaki et al., 2018), in comparison to lean mice, mT models in obese mice presented high metastatic dissemination to distant sites (Figures 2E,F and Supplementary Figure 2B), while no metastases were evident at 2 months in mice transplanted with preneoplastic organoids. However, we limited the study at mT models metastatization time-point, thus we cannot exclude a later metastatic onset for mP organoids in GIO and DIO models.

Overall, our mouse models confirmed that obesity not only promoted engraftment incidence rate and growth of both preneoplastic and neoplastic organoid models but also affected the pancreatic cancer progression measured as lesion growth, PanIN evolution and distant metastases dissemination. This model well fit with the human PDAC pathology, with a high number of obese patients presenting with metastasis and more aggressive PDAC at the diagnosis (Yuan et al., 2013; Abbruzzese et al., 2018).

### Obesity Induced a Change of Circulating Proinflammatory Signature and a Re-modulation of the Pancreatic Tissue Immune Infiltrate

Obesity is characterized by alterations in immune and inflammatory functions. In order to evaluate the potential role of cytokines expression in obesity-associated immunity, we analyzed immune cell composition in blood and tissue as well as circulating inflammatory cytokines profile of both mP and mT transplant models in DIO mice 35 days after orthotopic injection.

The cytofluorimetric analysis demonstrated that obesity affected both the total number and relative composition of myeloid and lymphoid components in the peripheral blood samples of our models.

**FIGURE 3:**
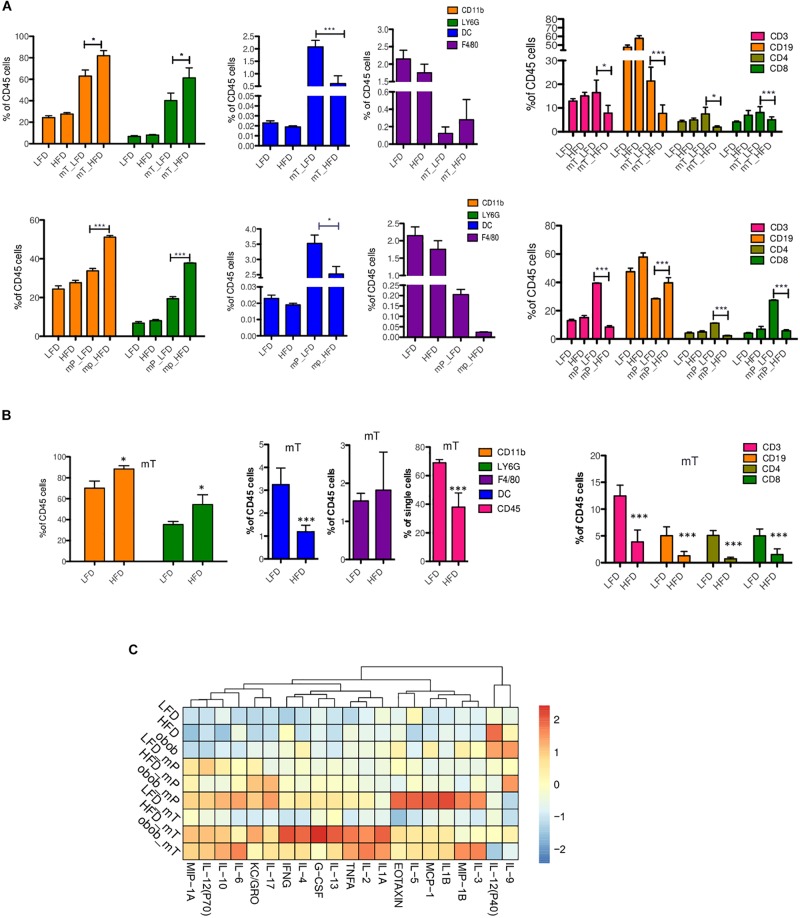
Immune and cytokine profiles of organoid-transplant model systems. Cytofluorimetric analysis for immune cells in **(A)** peripheral blood of mP and mT organoids model bearing-mice and **(B)** in the resected pancreata of mT organoids model bearing-mice. The data were shown as a percentage relative to the CD45^+^ cell population. Three independent experiments are shown; **(C)** Multiplex analysis of proinflammatory circulating factors. Plasma from peripheral blood was collected from each group of mice (*n* = 3) before and after (LFD *n* = 3, HFD *n* = 3, Ob/Ob *n* = 2) (35 days) mP and mT organoid injections. Concentrations of proinflammatory cytokines were analyzed using Luminex technology. Concentrations of cytokines (pg/mL) were calculated. The values for each cytokine (chemokine was reported as fold increase of each protein respect to expression value of mP LFD model as control. The mean values and standard deviation are shown. ***, *p* < 0.001; *, *p* < 0.05 by two-tailed unpaired Student’s *t*-tests.

In detail, we found that CD11b^+^-expressing cells expanded significantly in peripheral blood samples of DIO mice of both mT and mP *in vivo* models. Organoids transplant models of obese mice compared to lean mice showed a statistically significant increase of CD11b^+^Ly6G^+^ (polymorphonuclear cells, PMN) with a concomitant reduction of CD11b^+^CD11c^+^ (dendritic cells, DC), CD3^+^ (T lymphocytes) and CD19^+^ (B lymphocytes) circulating cells (Figure 3A).

To confirm that the presence of adipose tissue altered the dynamics of immune infiltration in our models, we analyzed the pancreata of mT models. In the pancreatic tissue from mT-LFD models, CD45^+^ cells represented the 69,25 ± 1,62% of total cells whereas a decrease was observed in obese models (38,76% ± 19,6%). As expected, the nature of mT neoplastic tissue infiltrating cells was different in obese compared to lean mice model reflecting the reduction of inflammatory anti-tumor cell subsets. Indeed, pancreatic tissue of mT obese models showed a reduction of both T lymphocyte and DC cells compared to lean mice (Figure 3B). The high percentage of infiltrating T lymphocytes, in particular of CD8^+^ T cells, has been associated to an increase of PDAC patients overall survival (Balachandran et al., 2017). Therefore, we sought to assess the degree of infiltrating lymphocytes in our DIO models of pancreatic carcinogenesis and found a statistically significant reduction of infiltrating CD3-and CD8- expressing cells, and a trend toward the reduction of CD4^+^ T cells in HFD compared to LFD models (Figure 3B).

Overall, these results suggest that pancreatic obesity-induced carcinogenesis is associated to accumulation of a myeloid infiltrate, with granulocytes becoming prominent in preinvasive lesions of obese models compared to lean models, while T cells are excluded as tumor progresses. These changes in immune infiltration of the tumor were associated to specific changes in circulating cytokines/chemokines, with an increased level of G-CSF and a subset of anti-inflammatory Th2 cytokine (IL-6 and IL-10) in pancreatic lesions of obese models compared to lean models (Figure 3C). Interestingly, we also found an increase of circulating levels of IL-17 in obese compared to lean mice models (Figure 3C). Previous studies have demonstrated that IL-17 produced by infiltrating immune cells is necessary for initiation and progression of PanIN (McAllister et al., 2014) and that IL-10 secreted by γδ Treg cells diminished the cytotoxic activity of CD8^+^ T cells and NK cells, resulting in tumor growth (Seo et al., 1998).

We also evaluated the Th1, Th2, and Th17 cytokines expression profile in our models (Figure 3C). We found a slight increase of tumor initiating cytokines such as TNFα, IL-17, and IL-13 in mP obese models, while a statistically significant increase in the more aggressive mT obese models. Furthermore, the cytokines related to tumor growth, EMT, metastasis and drug resistance, such as IL-1B, IL-2, Il-4, IL-6, IL-10, IL-17, KC (GRO), were mainly expressed in mT obese models.

**FIGURE 4:**
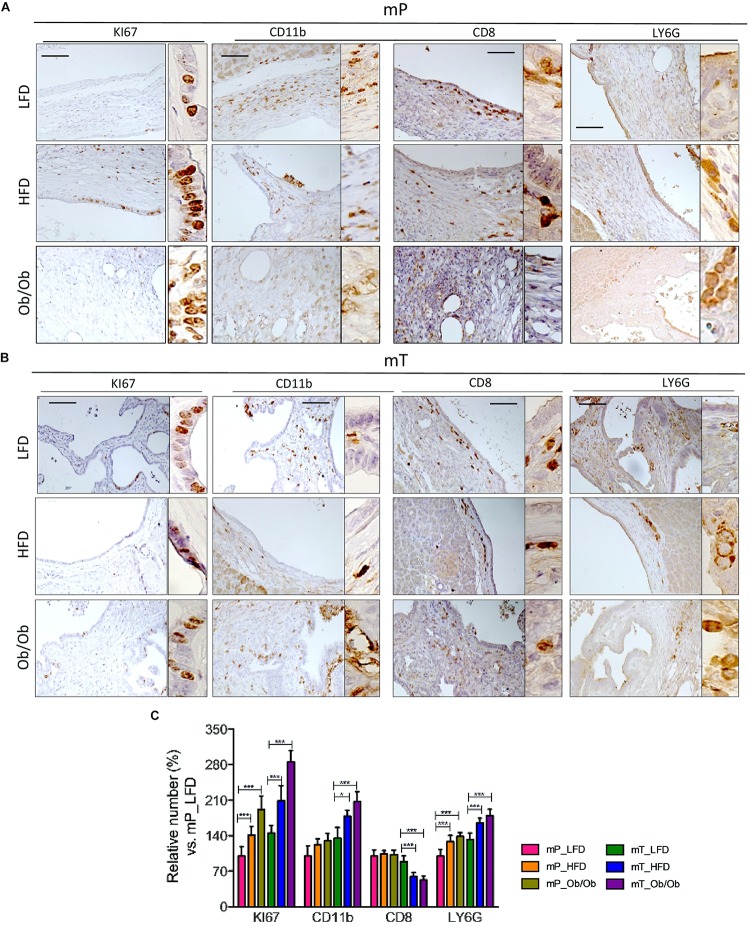
Obesity affected the immune infiltrate in organoid –transplant systems. Representative immunohistochemical staining for Ki67, CD11b, CD8, and LY6G positive cells in tissues from mice bearing **(A)** mP and **(B)** mT organoid-derived cells. Scale bars, 100 μm. The experiment was performed in three model of each group. **(C)** Quantification of paraffin-embedded tumor sections stained immunohistochemically with antibodies against Ki67, CD11b, CD8, and LY6G. Number of positive cells were from ten different area with pancreatic lesions. The mean values and standard deviation are shown. ***, *p* < 0.001; *, *p* < 0.05 by two-tailed unpaired Student’s *t*-tests.

Immunohistochemical staining of Ki67^+^ cell growth marker, demonstrated that obesity increased the proliferation of both mP and mT organoid-derived cells. Moreover IHC analysis of pancreata from mP and mT obese models confirmed the increase of a protumoral immune cell composition with an expansion of LY6G^+^-MDSC cells in the pancreatic tissue of obese respect to lean models, and a concomitant statistically significant reduction of CD8+ infiltrating cells (Figure 4).

Taken together these data confirmed that obesity accelerates infiltration of immunosuppressive environment mimicking human pancreatic carcinogenesis.

### Obesity Sustains a Specific Transcriptional Profile During Pancreatic Carcinogenesis

To better understand the effect of adipose tissue on pancreatic carcinogenesis, we performed a comparison between transcriptomes of GIO, DIO, and lean mT mice; the same analysis has not been performed on samples derived from mP models, because of the small amount of mP LFD derived tissue (as shown in Figure 1C).

**FIGURE 5:**
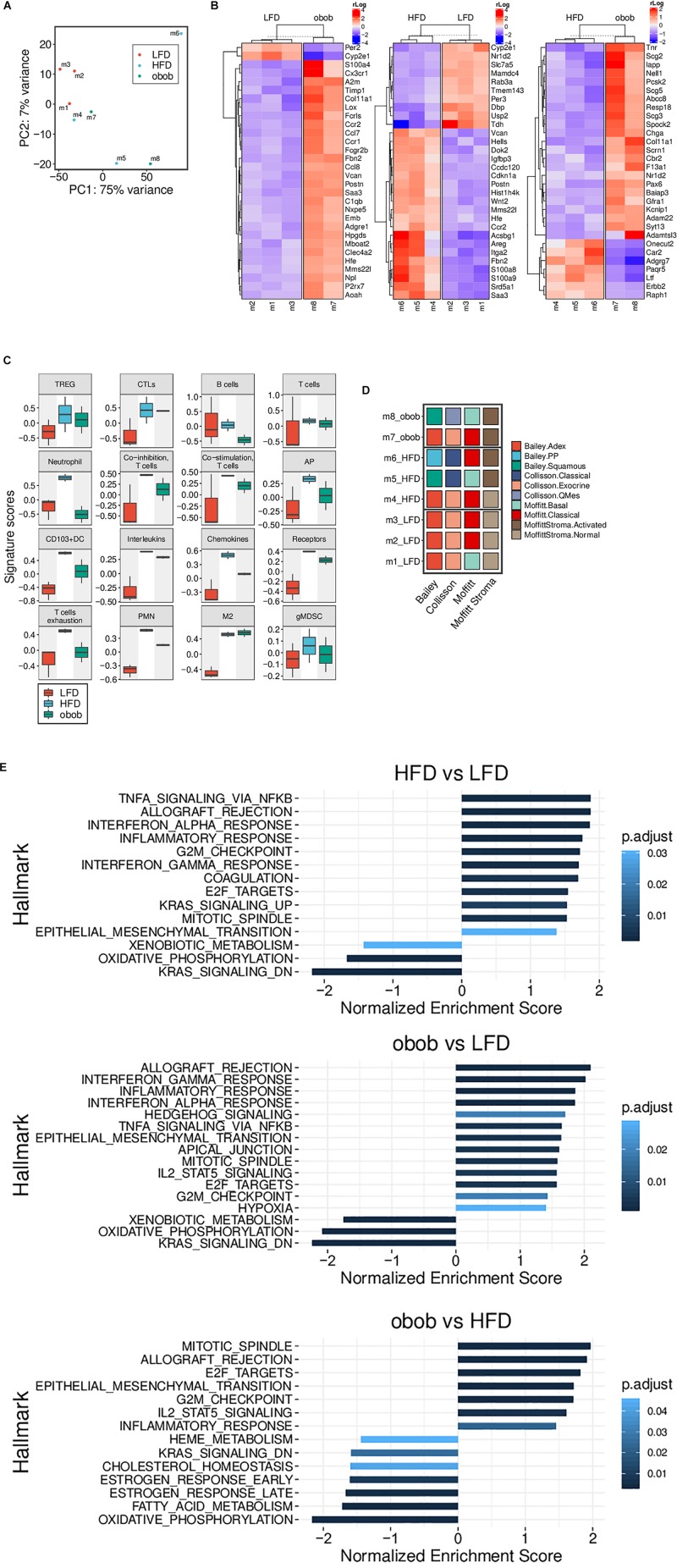
Obesity-induced carcinogenesis and progression of mT organoids models are associated with changes of gene expression profile. **(A)** RNA-Seq based PCA showing a homogeneous cluster of LFD mice, while HFD and ob/ob ones display a more heterogeneous profile. **(B)** Heatmap of the differentially expressed genes from mT of obese and lean mice. Top 30 genes with an adjusted *p*-value < 0.05 are reported. **(C)** Boxplots showing the RNA-Seq based GSVA scores of different custom gene sets (LFD, *n* = 3, HFD, *n* = 3, ob/ob *n* = 2). **(D)** The panel represents the RNA-Seq based subtyping for each individual mT mice of the three groups. A sample was assigned to a Bailey, Collisson or Moffitt (Stroma subtypes separated) subtype when its GSVA score was highest within that subtype group. LFD mice are clearly more homogenous than HFD and ob/ob. **(E)** Results of the pre-ranked GSEA applied to the results of the differential gene expression between the three pairs indicated. Only gene sets with an adjusted *p*-value < 0.05 are displayed.

RNA-seq based Principal Component Analysis (PCA) showed that only LFD mice cluster homogeneously, whereas both HFD and ob/ob display a heterogeneous profile (Figure 5A). In addition, we found clusters of genes differentially expressed in LFD compared to both ob/ob and HFD and HFD compared to ob/ob (Figure 5B).

In particular, Gene Set Variation Analysis (GSVA) (Figure 5C) confirmed an increase of expression of genes related to immune-tolerance or immune suppression, such as IL-1A and B, IL-4, IL-6, and IL-10 (confirmed also by luminex assay, Figure 3C), in mT from obese models compared to lean mT mice models.

Indeed, mT organoids from obese compared to lean models, showed an up-regulation of M2 and MDSC related genes that could support growth and progression; these data are supported by the cytofluorimetric analysis performed on both peripheral blood and tumor tissue, IHC and Luminex assay.

In order to explain the contribution of obesity in pancreatic carcinogenesis, we categorized mT derived tumors according to different available classification for pancreatic cancer (Waddell et al., 2015; Bailey et al., 2016; Collisson et al., 2019) and stroma (Moffitt et al., 2015; Ohlund et al., 2017; Whittle and Hingorani, 2019). As shown in Figure 5D, the mT from LFD displayed a less aggressive behavior and a “normal” stroma, whereas both HFD and ob/ob groups have a heterogeneous distribution among the various subtypes and they are able to induce stromal activation, suggesting a role for obesity in this process. Furthermore, Gene Set Enrichment Analysis (GSEA) of mT from GIO and DIO mice showed a significant up-regulation of the NFkB, TNF-alpha and epithelial to mesenchymal transition (EMT) pathways and confirmed the up-regulation of cytokine signaling pathways compared to lean models (Figure 5E). In addition, mT from obese mice showed up-regulation of basement membranes cell adhesion, proliferation, migration, angiogenesis and tissue morphogenesis maintenance pathways and a down regulation of KRAS activated genes and oxidative phosphorylation pathway.

Altogether, these data support the previously shown IHC and cytofluorimetric analysis and point out a role for obesity in neoplastic expansion, progression and metastatization as indicated by the up-regulation of pathways related to these processes; it is still unclear if this ability is due to a direct influence of adipose tissue on tumor intrinsic signaling pathways or to the activation of inflammatory processes that leads to the establishment of an immunosuppressive environment which promotes tumor progression.

### Analysis of Circulating Factors During Obesity–Induced Pancreatic Carcinogenesis

**FIGURE 6:**
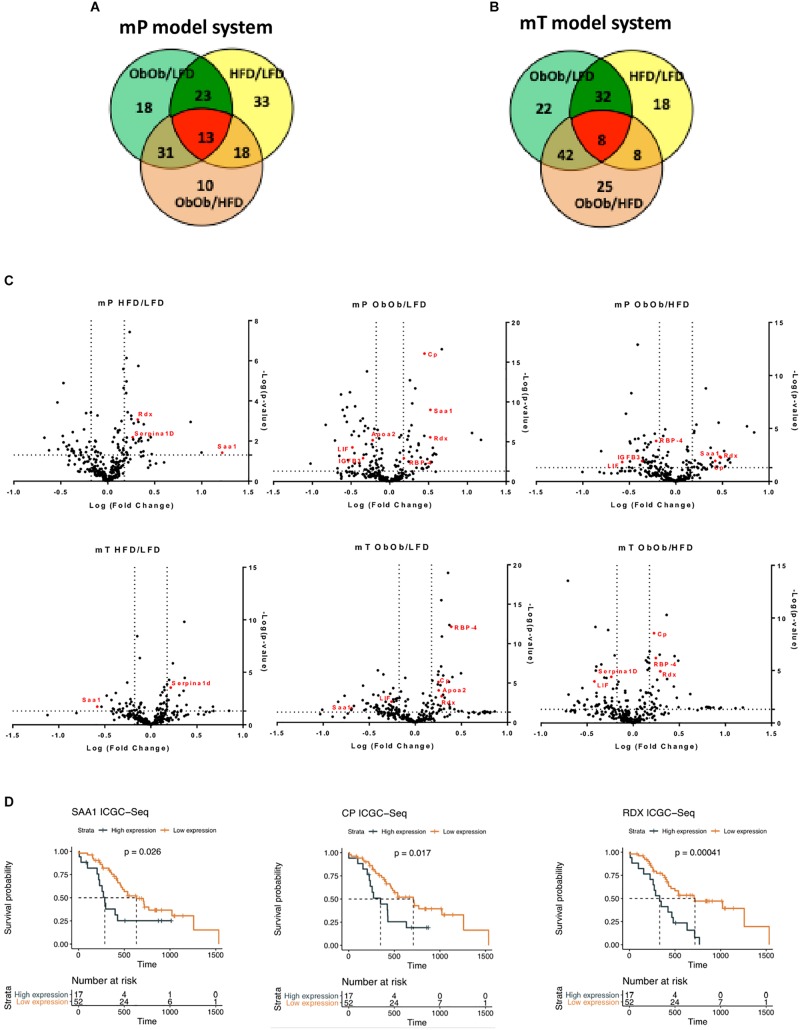
Proteomic analysis from DIO, GIO and LFD mP and mT tumor-bearing mice confirmed the increase of inflammation and carcinogenesis in obese mouse. Venn diagram of modulated proteins in mP **(A)** and mT **(B)** model systems. **(C)** Volcano plot showing differentially expressed proteins. The volcano plot showed the results of differentially expressed proteins systems based on fold change versus *t*-test probability from both mP and mT pancreatic organoids in obese mice rispect to lean mice. Each protein is represented as a dot and is mapped according to its fold change on the ordinate axis (*y*) and *t*-test *p*-value on the abscissa axis (*x*). **(D)** Survival analyses of pancreatic cancer patients dichotomized by high and low expression of some of differentially upregulated proteins in the serum of obese models rispect to lean mice. The overall survival data were obtained from https://dcc.icgc.org/releases/current/Projects/PACA-AU. Median survival was estimated with the Kaplan-Meier method and the difference was tested using the log-rank (Mantel-Cox) test.

To demonstrate that the proposed organoid model systems are an accurate and precise model of pancreatic carcinogenesis, we analyzed circulating proteins into the plasma of our organoids model systems. We successfully characterized differential proteomic profiles of plasma from DIO, GIO and LFD mP and mT tumor-bearing mice. A total of 290 proteins were quantified in all the samples. The proteomic analysis confirmed an increase of obesity, metabolism and inflammatory activated pathways in obese respect to the lean models, as well as several differentially regulated proteins involved in pancreatic carcinogenesis. In particular, we identified 36 and 40 circulating proteins that were differentially expressed (fold change >1.5 and *p*-value < 0.05) by mP an mT obese models, respectively (Figures 6A,B and Supplementary Table 1). Several of these proteins are already described to be involved in PDAC raising our organoid-transplant model systems as highly significant biologic models for the rapid and precise reproduction of pancreatic carcinogenesis.

For example, apolipoprotein A2 (ApoA2) was one of the top obesity-regulated proteins. It has been recently demonstrated, by plasma analysis of European EPIC cohort patients, that the combination CA19-9 and ApoA2 may improve detection of pancreatic cancer compared to CA19-9 alone (Honda et al., 2019). Also a significant elevation in serum concentrations of RBP-4 (Noy et al., 2015) and of Serpina1d (Elwakeel et al., 2019) was found in pancreatic cancer patients. In addition, it has been demonstrated that LIF was overexpressed in tumor tissue compared with healthy pancreas. Moreover, LIF has been candidate as serum biomarker and diagnostic tool for PDAC metastatic progression (Bressy et al., 2018). Along with pancreatic cancer progression, cachexia is a common event (Henderson et al., 2018). In our model, insulin-like growth factor-binding protein 3 (IGFB3), a protein able to induce muscle wasting, was upregulated (Rohrmann et al., 2012). Interestingly, peroxisome proliferator-activated receptors (PPARs) signaling pathway resulted activated in obese mice: PPARs are nuclear hormone receptors which play an important role in regulating cancer cell proliferation, survival, apoptosis, and tumor growth. PPAR is a key regulator of adipocytes differentiation, it regulates insulin and adipokines production and secretion and may modulate inflammation (Siersbaek et al., 2010). Moreover, PPAR was found upregulated in other human malignancies, including pancreatic cancer, where its upregulation is correlated with higher pathological grade and increased risk of metastasis (Eibl, 2008). To validate our model systems, the most significant differentially expressed factors secreted along pancreatic cancer progression, were tested on the data of world’s largest repository of cancer sequencing of the International Cancer Genome Consortium (ICGC). These *in silico* analyses showed that patients with an high expression of some of the genes [Radixin (RDX), Ceruloplasmin (CP), and Serum amyloid A1 (SAA1)] that we found upregulated in our model, have a worse prognosis than patients with low expression of the same factors (Figures 6C,D).

## Discussion

Obesity is considered to be one of the most important risk factors for PDAC and many cohort studies highlighted the strong correlation between obesity and pancreatic cancer incidence (Davoodi et al., 2013; Font-Burgada et al., 2016; Cascetta et al., 2018). Although genetic events responsible for pancreatic carcinogenesis (Winter et al., 2006; Reiter and Iacobuzio-Donahue, 2016) and early dissemination processes were well established (Rhim et al., 2012), the effects of obesity on these processes are still unknown.

Several data suggested different molecular mechanisms whereby obesity leads to higher incidence of cancer such as increased oxidative stress, hormonal disorder, dysbiosis, and chronic inflammation (Bianchini et al., 2002; Poloz and Stambolic, 2015; Font-Burgada et al., 2016; Donohoe et al., 2017; Sasaki et al., 2018).

However, how obesity influences the early step of carcinogenesis, including immune cells trafficking, remains largely elusive.

This is mainly due to the use of models that already retain all cancer characteristics to quickly evolve in an aggressive disease course. In this regard, Incio et al. (2016) demonstrated that obesity-induced inflammation and neutrophils infiltration lead to a desmoplastic tumor microenvironment, which directly promotes tumor growth and impairs the response to chemotherapy.

Sasaki et al. (2018), using a cell competition *in vivo* model, demonstrated that obesity and chronic inflammation influence cell competition within the epithelium of several organs, including pancreas. These interesting results are limited to the observation of an increase of cell competition in lean models suggesting that non-steroidal anti-inflammatory drugs (NSAIDs) can suppress the frequency of tumor formation. Indeed, has been reported, in two different patients’ cohorts, also the role of leptin in increasing pancreatic cancer risk (Stolzenberg-Solomon et al., 2015; Babic et al., 2016).

However, the underlying molecular mechanisms remain unknown. Recently, it has been investigated the role of a metabolic regulator that prevents obesity, fibroblast growth factor 21 (FGF21), in KRAS oncogenic context of pancreatic cancer. It has been demonstrated that FGF21 has the potentiality to reduce the effects of obesity on pancreatic carcinogenesis (Luo et al., 2019).

Although these models offered a high potential for the study of pancreatic cancer, including investigation of therapeutic strategies and putative mechanisms of resistance, they are often affected by several consistent gaps.

Transplantation of pancreatic isogenic cancer cell lines for example, are so far distant from reproducing a precise model of carcinogenesis. This is due to the ability of already established cancer cells to grow also in the absence of external stimuli. Further, in these models, pancreatic cancer is a very aggressive disease with a rapid cancer growth that excludes the influence of tumor microenvironment.

So on, genetically engineered mouse models that spontaneously develop pancreatic cancer, although were precise models of pancreatic carcinogenesis, often presented a variable intra-experimental delay in the starting time of carcinogenesis, rendering these models less suitable to study the influence of adipose tissue.

Here we propose, to our knowledge, the best suitable and manageable model linking obesity to pancreatic carcinogenesis using organoid transplant systems. Here, we demonstrated that both DIO and GIO models promoted engraftment incidence rate and growth of both preneoplastic and neoplastic organoid models, favoring the pancreatic cancer progression measured as lesion growth, PanIN evolution and distant metastases dissemination.

However, we recognize that GIO models are not the best tools, being leptin deficient and showing an obesity that does not reflect the characteristics of pancreatic cancer patients. They have been a useful comparison model and served for a more complete picture of the pancreatic cancer acceleration of mP and mT DIO models.

Our data suggested a model in which obesity mimics the evolution of human pancreatic cancer pathology, promoting carcinogenesis concomitant to the accumulation of a myeloid infiltrate, and exclusion of T cells as tumor progresses. The changes in cancer immune infiltrate were associated with specific changes in circulating cytokines/chemokines, with an increased level of a subset of anti-inflammatory Th2 cytokines in pancreatic lesions of obese models compared to lean models. Analysis of the gene expression profile of neoplastic cells in different conditions, revealed a change in pancreatic cancer subtype under obesity conditions as well as an activation of stromal cells that have been reported to play a crucial role in PDAC development and progression (Moffitt et al., 2015).

Overall, here we propose organoid-based model systems to study *in vivo* the effects of obesity on the pancreatic carcinogenesis. However, this work is limited to the observation of the potential obesity-related mediators of carcinogenesis and further functional analyses are required to validate each identified molecule.

## Data Availability Statement

Proteomic Data Availability: Data are available via ProteomeXchange with identifier PXD018362. RNAsequencind Data availability: The RNAsequencing (record GSE148135) data are available at https://www.ncbi.nlm.nih.gov/geo/query/acc.cgi?acc=GSE148135.

## Ethics Statement

The animal study was reviewed and approved by the University of Verona Animal Ethic Committee; CIRSAL approval number 1002/2016-PR.

## Author Contributions

FL: organoids generation and characterization. GP: organoids transplantation, multiplex analyses, tissue sampling, and RNA extraction. LT: obese mouse models generation and ultrasound imaging. PD: RNAseq and proteomic analysis. RT, AF, and FD: cytofluorimetric analysis. RB: immunohistochemistry and pancreatic histopathological analysis. DF: organoids generation and transplantation. MMan and EM: proteomic analysis. RTL: tissue sampling and management. MMar: tissue sampling, IHC and data analysis, manuscript finalization. GT: concept and design, data interpretation, and manuscript finalization. SU and VC: data interpretation, writing team, and manuscript finalization. DM: concept and design, data interpretation, and manuscript finalization. CC: concept and design, data interpretation, writing team, and manuscript finalization.

## Conflict of Interest

The authors declare that the research was conducted in the absence of any commercial or financial relationships that could be construed as a potential conflict of interest.

## Footnotes

1 https://www.ncbi.nlm.nih.gov/geo/

## Supplementary Material

FIGURE S1 Generation of an obese syngeneic model of pancreatic cancer progression. **(A)** Representative picture of obesity mouse models for pancreatic cancer progression study. **(B)** Time course of weight gain in High-fat diet (HFD, 60%) and leptin deficient (ob/ob) versus low-fat diet (LFD, 10%). The indicated red line was the time-point of organoid models’ injection. Histograms representing the gain of **(C)** body weight and **(D)** the width of the flank of the indicated group of mice. ***, *p* < 0.001.

FIGURE S2 Representative picture of **(A)** excised tumors and spleen from the indicated model systems and **(B)** of lung and liver metastasis.

FIGURE S3 Representative immunohistochemical staining for MUC5AC and 1685 MUC6 in tissues from mice bearing mP **(A)** and mT **(B)** organoid-derived cells. Scale 1686 bars, 50 μm. The experiment was performed in three model of each group.

TABLE S1 Circulating proteins differentially expressed by mP and mT obese respect to lean mice models.
