# Supplementary figures and images for: Organoid-Transplant Model Systems to Study the Effects of Obesity on the Pancreatic Carcinogenesis in vivo
Source: Front Cell Dev Biol. 2020 Apr 28;8:308. doi: 10.3389/fcell.2020.00308 (PMC7198708; doi:10.3389/fcell.2020.00308)

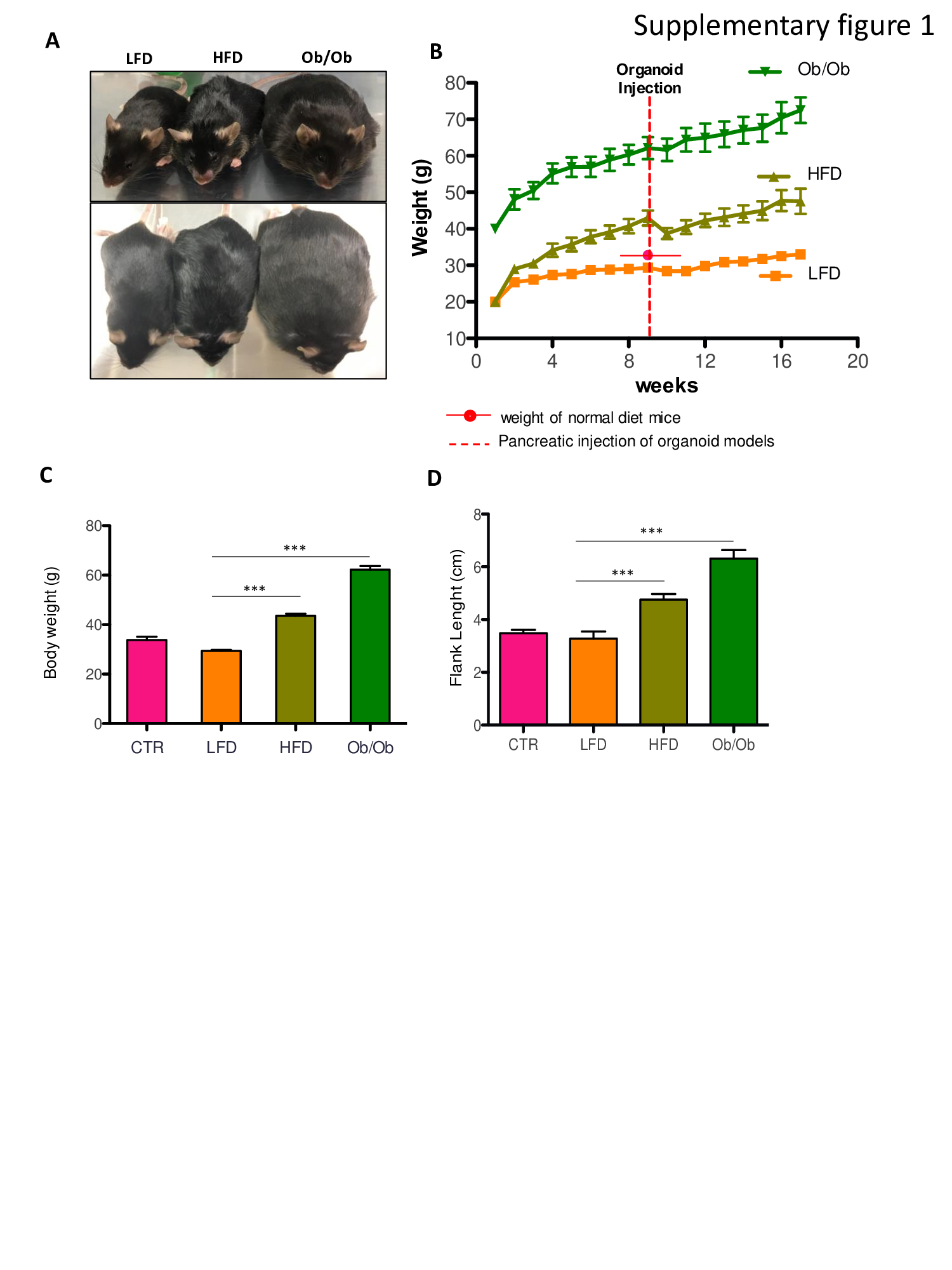

Supplement: FIGURE S1 — Generation of an obese syngeneic model of pancreatic cancer progression. (A) Representative picture of obesity mouse models for pancreatic cancer progression study. (B) Time course of weight gain in High-fat diet (HFD, 60%) and leptin deficient (ob/ob) versus low-fat diet (LFD, 10%). The indicated red line was the time-point of organoid models’ injection. Histograms representing the gain of (C) body weight and (D) the width of the flank of the indicated group of mice. ***, p < 0.001. [file Image_1.JPEG]

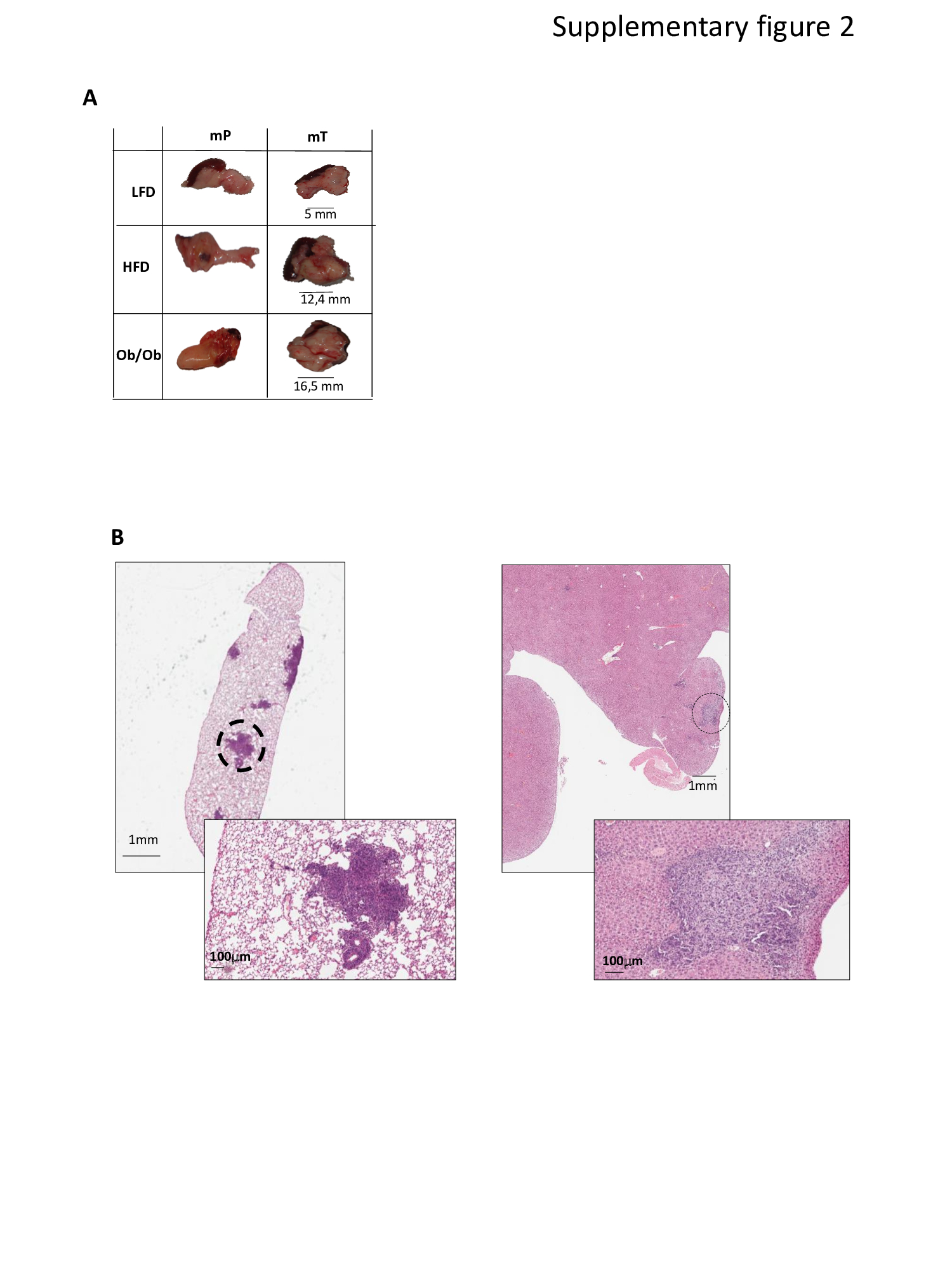

Supplement: FIGURE S2 — Representative picture of (A) excised tumors and spleen from the indicated model systems and (B) of lung and liver metastasis. [file Image_2.JPEG]

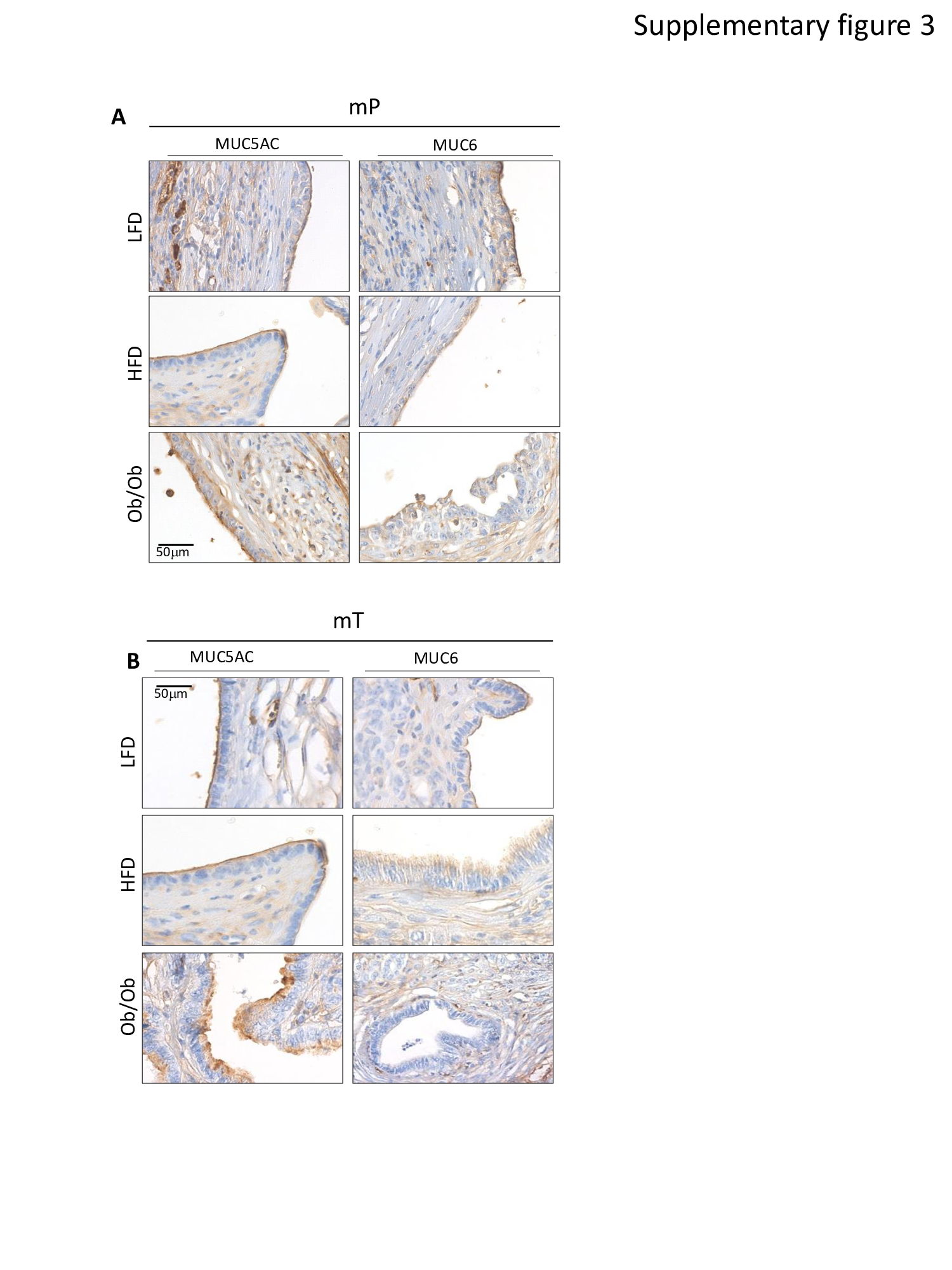

Supplement: FIGURE S3 — Representative immunohistochemical staining for MUC5AC and 1685 MUC6 in tissues from mice bearing mP (A) and mT (B) organoid-derived cells. Scale 1686 bars, 50 μm. The experiment was performed in three model of each group. [file Image_3.JPEG]
