# Supplementary material for: Organoid-Transplant Model Systems to Study the Effects of Obesity on the Pancreatic Carcinogenesis in vivo
Source: Front Cell Dev Biol. 2020 Apr 28;8:308. doi: 10.3389/fcell.2020.00308 (PMC7198708; doi:10.3389/fcell.2020.00308)
Supplement: TABLE S1 — Circulating proteins differentially expressed by mP and mT obese respect to lean mice models. [file Table_1.PDF]

Supplementary Table 1. Circulating proteins differentially expressed by mP and mT obese respect to lean mice models.

	Gene Symbol	Models	Role in pancreatic cancer	References
1	Ighg1	mT-ObOb/LFD and HFD/LFD	The presence of IGHG1 in human pancreatic carcinomas is associated with immune evasion mechanisms.	(Li et al., 2011)
2	Smarca4/BRG1	mT-ObOb/LFD and HFD/LFD	The BRG1/SOX9 axis is critical for acinar cell-derived pancreatic tumorigenesis.	(Tsuda et al., 2018)
3	Lifr	mT-ObOb/LFD and HFD/LFD and ObOb/HFD	Targeting LIF-mediated paracrine interaction for pancreatic cancer therapy and monitoring;<br>Leukemia inhibitory factor receptor negatively regulates the metastasis of pancreatic cancer cells in vitro and in vivo.	(Ma et al., 2016; Shi et al., 2019)
4	Plg	mT-ObOb/LFD and HFD/LFD	Soluble stroma-related biomarkers of pancreatic cancer. "we found three markers, FN, Col4, and PLG, significantly different in PDAC versus pancreatitis. The combination of these three markers with CCN2 and CA19.9 had a significant capability to discriminate between PDAC and CP."	(Resovi et al., 2018)
5	Apoc2	mP-, mT-ObOb/LFD and HFD/LFD	Serum apolipoprotein C-II is prognostic for survival after pancreatic resection for adenocarcinoma.	(Xue et al., 2012)
6	Selenop	mT-ObOb/LFD and HFD/LFD	Selenoprotein P, as a predictor for evaluating gemcitabine resistance in human pancreatic cancer cells.	(Maehara et al., 2004)
7	Cp	mP-ObOb/LFD and HFD/LFD and ObOb/HFD	Secretome protein signature of human pancreatic cancer stem-like cells. "ceruloplasmin as promising marker for patients negative for CA19-9. "	(Brandi et al., 2016)
8	S100a6	mT-ObOb/LFD and HFD/LFD	S100 calcium-binding protein A6 promotes epithelial-mesenchymal transition through β-catenin in pancreatic cancer cell line.	(Chen et al., 2015)
9	Lum	mP-ObOb/LFD and HFD/LFD	Prolonged exposure to extracellular lumican restrains pancreatic adenocarcinoma growth.	(Li et al., 2017)
10	Ahsg	mT-ObOb/LFD and HFD/LFD	Profiling the potential tumor markers of pancreatic ductal adenocarcinoma using 2D-DIGE and MALDI-TOF-MS: up-regulation of Complement C3 and alpha-2-HS-glycoprotein.	(Chen et al., 2013a)
11	Asap2 or centaurin	mP-ObOb/LFD and HFD/LFD	Profiling the potential biomarkers for cell differentiation of pancreatic cancer using iTRAQ and 2-D LC-MS/MS.	(Zhang et al., 2009)
12	Itih2	mT-ObOb/LFD and HFD/LFD	Plasma extracellular vesicle long RNA profiling identifies a diagnostic signature for the detection of pancreatic ductal adenocarcinoma	(Yu et al., 2019)
13	Apoa2	mT-ObOb/LFD and HFD/LFD	CA19-9 and apolipoprotein-A2 isoforms as detection markers for pancreatic cancer: a prospective evaluation.<br>A simplified diagnostic panel of CA19-9, ApoC-I and ApoA-II improves the diagnostic ability of CA19-9 alone and may have clinical utility.<br>Plasma biomarker for detection of early stage pancreatic cancer and risk factors for pancreatic malignancy using antibodies for apolipoprotein-AII isoforms.	(Honda et al., 2015) (Honda et al., 2019) (Xue et al., 2010)
14	Apoa4	mT-ObOb/LFD and HFD/LFD and ObOb/HFD	Large-scale clinical validation of biomarkers for pancreatic cancer using a mass spectrometry-based proteomics approach.	(Park et al., 2017b)
15	Orm2 or AGP-2	mP-ObOb/LFD and HFD/LFD and ObOb/HFD	Increased α1-3 fucosylation of α-1-acid glycoprotein (AGP) in pancreatic cancer.	(Balmana et al., 2016)
16	Tmsb4x or Thymosin beta-4	mT-ObOb/LFD and HFD/LFD	In situ proteomic analysis by MALDI imaging identifies ubiquitin and thymosin-β4 as markers of malignant intraductal pancreatic mucinous neoplasms.	(Rebours et al., 2014)
17	Cfh	mP-ObOb/LFD and HFD/LFD	Identification of potential prognostic biomarkers in patients with untreated, advanced pancreatic cancer from a phase 3 trial (Cancer and Leukemia Group B 80303).	(Roberts et al., 2012)
18	Saa1	mP, mT-ObOb/LFD and HFD/LFD	Hepatocytes direct the formation of a pro-metastatic niche in the liver. "During early pancreatic tumorigenesis in mice, hepatocytes show activation of signal transducer and activator of transcription 3 (STAT3) signalling and increased production of serum amyloid A1 and A2 (referred to collectively as SAA). Overexpression of SAA by hepatocytes also occurs in patients with pancreatic and colorectal cancers that have metastasized to the liver, and many patients with locally advanced and metastatic disease show increases in circulating SAA."	(Lee et al., 2019)
19	Saa2	mP, mT-ObOb/LFD and HFD/LFD	Hepatocytes direct the formation of a pro-metastatic niche in the liver. "During early pancreatic tumorigenesis in mice, hepatocytes show activation of signal transducer and activator of transcription 3 (STAT3) signalling and increased production of serum amyloid A1 and A2 (referred to collectively as SAA). Overexpression of SAA by hepatocytes also occurs in patients with	(Lee et al., 2019)

			pancreatic and colorectal cancers that have metastasized to the liver, and many patients with locally advanced and metastatic disease show increases in circulating SAA."	
20	ApoC1	mT-ObOb/LFD and HFD/LFD	The specific expression of ApoC-1 and its role in preventing from spontaneous apoptosis in pancreatic cancer cells suggest that ApoC-1 contributes to the aggressiveness of pancreatic cancer and will be useful as a new therapeutic target.	(Takano et al., 2008)
21	Hpx	mP-ObOb/LFD and HFD/LFD	Glycoprotein microarrays with multi-lectin detection: unique lectin binding patterns as a tool for classifying normal, chronic pancreatitis and pancreatic cancer sera.	(Zhao et al., 2007)
22	Serpina3m	mT-ObOb/LFD and HFD/LFD	Genetic Effects and Modifiers of Radiotherapy and Chemotherapy on Survival in Pancreatic Cancer	(Zeng et al., 2011)
23	Tmsb10	mT-ObOb/LFD and HFD/LFD	Gene expression analysis of pancreatic cell lines reveals genes overexpressed in pancreatic cancer. "By means of immunohistochemistry we could show that thymosin beta-10 (TMSB10), upregulated in tumor cell lines, is expressed in human pancreatic carcinoma, but not in non-neoplastic pancreatic tissue, suggesting a role for TMSB10 in the carcinogenesis of pancreatic carcinoma."	(Alldinger et al., 2005)
24	C3	mT-ObOb/LFD and HFD/LFD	Expression and clinical significance of complement C3, complement C4b1 and apolipoprotein E in pancreatic cancer.	(Chen et al., 2013b)
25	Serpina1d or alpha-1-trypsin	mT-ObOb/LFD and HFD/LFD and ObOb/HFD	Evidence of Altered Glycosylation of Serum Proteins Prior to Pancreatic Cancer Diagnosis.	(Krishnan et al., 2017)
26	Ttr	mP-ObOb/LFD and HFD/LFD	Diagnostic performance enhancement of pancreatic cancer using proteomic multimarker panel.	(Park et al., 2017a)
27	Serping1 or plasma protease C1 inhibitor	mP-ObOb/LFD and HFD/LFD	Comparative serum glycoproteomics using lectin selected sialic acid glycoproteins with mass spectrometric analysis: application to pancreatic cancer serum. "Sialylated plasma protease C1 inhibitor is identified to be down-regulated in cancer serum."	(Zhao et al., 2006)
28	Rbp4	mT-ObOb/LFD and HFD/LFD and ObOb/HFD	Clinical value of circulating lipocalins and insulin-like growth factor axis in pancreatic cancer diagnosis.	(El-Mesallamy et al., 2013)
29	Smarca4 or BRG1	mT-ObOb/LFD and HFD/LFD	BRG1 promotes chemoresistance of pancreatic cancer cells through crosstalking with Akt signalling.	(Liu et al., 2014)
30	B2m	mT-ObOb/LFD and HFD/LFD	Beta 2-microglobulin regulates amyloid precursor-like protein 2 expression and the migration of pancreatic cancer cells	(Sliker et al., 2019)
31	Hp	mP-ObOb/LFD and HFD/LFD	Application of glycoscience to the early detection of pancreatic cancer.	(Miyoshi and Kamada, 2016)
32	Apoa1	mP-ObOb/LFD and HFD/LFD and ObOb/HFD	Apolipoprotein A-I mimetic peptide 4F suppresses tumor-associated macrophages and pancreatic cancer progression.	(Peng et al., 2017)
33	ApoC1	mP-ObOb/LFD and HFD/LFD	A simplified diagnostic panel of CA19-9, ApoC-I and ApoA-II improves the diagnostic ability of CA19-9 alone and may have clinical utility.	(Xue et al., 2010)
34	Apoe	mP, mT-ObOb/LFD and HFD/LFD	A new panel of pancreatic cancer biomarkers discovered using a mass spectrometry-based pipeline. "The novel biomarker panel of apolipoprotein E (APOE), inter-alpha-trypsin inhibitor heavy chain H3 (ITIH3), apolipoprotein A-I (APOA1), apolipoprotein L1 (APOL1), combining with CA19-9, statistically-significantly improved the sensitivity (95%) and specificity (94.1%), outperforming CA19-9 alone, for the diagnosis of PC."	(Liu et al., 2017)
35	C8a	mT-ObOb/LFD and HFD/LFD and ObOb/HFD	N/A	N/A
36	Itih1	mT-ObOb/LFD and HFD/LFD	N/A	N/A
37	Cpn1	mT-ObOb/LFD and HFD/LFD	N/A	N/A
38	Apoc4	mT-ObOb/LFD and HFD/LFD	N/A	N/A
39	F13a1	mT-ObOb/LFD and HFD/LFD	N/A	N/A
40	1300017J02Rik	mT-ObOb/LFD and HFD/LFD	N/A	N/A
41	Cpn2	mT-ObOb/LFD and HFD/LFD	N/A	N/A
42	Rims1	mT-ObOb/LFD and HFD/LFD	N/A	N/A
43	Pltp	mT-ObOb/LFD and HFD/LFD	N/A	N/A

44	Apoh	mT-ObOb/LFD and HFD/LFD	N/A	N/A
45	Apcs	mT-ObOb/LFD and HFD/LFD	N/A	N/A
46	C8g	mP-ObOb/LFD and HFD/LFD and ObOb/HFD	N/A	N/A
47	Hba	mT-ObOb/LFD and HFD/LFD and ObOb/HFD	N/A	N/A
48	C2	mP-ObOb/LFD and HFD/LFD	N/A	N/A
49	H2-Q10	mP-ObOb/LFD and HFD/LFD	N/A	N/A
50	Sec23ip	mP-ObOb/LFD and HFD/LFD	N/A	N/A
51	Dmxl2	mP-ObOb/LFD and HFD/LFD	N/A	N/A
52	Siae	mP-ObOb/LFD and HFD/HFD	N/A	N/A
53	Cpb2	mP-ObOb/LFD and HFD/LFD	N/A	N/A
54	Gp1ba	mP-ObOb/LFD and HFD/LFD	N/A	N/A
55	Apom	mP-ObOb/LFD and HFD/LFD and ObOb/HFD	N/A	N/A
56	Rdx	mP-ObOb/LFD and HFD/LFD and ObOb/HFD	N/A	N/A
57	Apod	mP-ObOb/LFD and HFD/LFD and ObOb/HFD	N/A	N/A
58	Serpinc1	mT-ObOb/LFD and HFD/LFD	N/A	N/A
59	Scgb1a1	mT-ObOb/LFD and HFD/LFD and ObOb/HFD	N/A	N/A
60	Serpina1e	mP-ObOb/LFD and HFD/LFD and ObOb/HFD	N/A	N/A

References.

Alldinger, I., Dittert, D., Peiper, M., Fusco, A., Chiappetta, G., Staub, E., et al. (2005). Gene expression analysis of pancreatic cell lines reveals genes overexpressed in pancreatic cancer. *Pancreatology* 5(4-5)**,** 370-379. doi: 10.1159/000086537.

Balmana, M., Gimenez, E., Puerta, A., Llop, E., Figueras, J., Fort, E., et al. (2016). Increased alpha1-3 fucosylation of alpha-1-acid glycoprotein (AGP) in pancreatic cancer. *J Proteomics* 132**,** 144-154. doi: 10.1016/j.jprot.2015.11.006.

Brandi, J., Dalla Pozza, E., Dando, I., Biondani, G., Robotti, E., Jenkins, R., et al. (2016). Secretome protein signature of human pancreatic cancer stem-like cells. *J Proteomics* 136**,** 1-12. doi: 10.1016/j.jprot.2016.01.017.

Chen, J., Wu, W., Chen, L., Zhou, H., Yang, R., Hu, L., et al. (2013a). Profiling the potential tumor markers of pancreatic ductal adenocarcinoma using 2D-DIGE and MALDI-TOF-MS: up-regulation of Complement C3 and alpha-2-HS-glycoprotein. *Pancreatology* 13(3)**,** 290-297. doi: 10.1016/j.pan.2013.03.010.

Chen, J., Wu, W., Zhen, C., Zhou, H., Yang, R., Chen, L., et al. (2013b). Expression and clinical significance of complement C3, complement C4b1 and apolipoprotein E in pancreatic cancer. *Oncol Lett* 6(1)**,** 43-48. doi: 10.3892/ol.2013.1326.

Chen, X., Liu, X., Lang, H., Zhang, S., Luo, Y., and Zhang, J. (2015). S100 calcium-binding protein A6 promotes epithelial-mesenchymal transition through beta-catenin in pancreatic cancer cell line. *PLoS One* 10(3)**,** e0121319. doi: 10.1371/journal.pone.0121319.

El-Mesallamy, H.O., Hamdy, N.M., Zaghloul, A.S., and Sallam, A.M. (2013). Clinical value of circulating lipocalins and insulin-like growth factor axis in pancreatic cancer diagnosis. *Pancreas* 42(1)**,** 149-154. doi: 10.1097/MPA.0b013e3182550d9d.

Honda, K., Katzke, V.A., Husing, A., Okaya, S., Shoji, H., Onidani, K., et al. (2019). CA19-9 and apolipoprotein-A2 isoforms as detection markers for pancreatic cancer: a prospective evaluation. *Int J Cancer* 144(8), 1877-1887. doi: 10.1002/ijc.31900.

Honda, K., Kobayashi, M., Okusaka, T., Rinaudo, J.A., Huang, Y., Marsh, T., et al. (2015). Plasma biomarker for detection of early stage pancreatic cancer and risk factors for pancreatic malignancy using antibodies for apolipoprotein-AII isoforms. *Sci Rep* 5, 15921. doi: 10.1038/srep15921.

Krishnan, S., Whitwell, H.J., Cuenco, J., Gentry-Maharaj, A., Menon, U., Pereira, S.P., et al. (2017). Evidence of Altered Glycosylation of Serum Proteins Prior to Pancreatic Cancer Diagnosis. *Int J Mol Sci* 18(12). doi: 10.3390/ijms18122670.

Lee, J.W., Stone, M.L., Porrett, P.M., Thomas, S.K., Komar, C.A., Li, J.H., et al. (2019). Hepatocytes direct the formation of a pro-metastatic niche in the liver. *Nature* 567(7747), 249-252. doi: 10.1038/s41586-019-1004-y.

Li, X., Kang, Y., Roife, D., Lee, Y., Pratt, M., Perez, M.R., et al. (2017). Prolonged exposure to extracellular lumican restrains pancreatic adenocarcinoma growth. *Oncogene* 36(38), 5432-5438. doi: 10.1038/onc.2017.125.

Li, X., Ni, R., Chen, J., Liu, Z., Xiao, M., Jiang, F., et al. (2011). The presence of IGHG1 in human pancreatic carcinomas is associated with immune evasion mechanisms. *Pancreas* 40(5), 753-761. doi: 10.1097/MPA.0b013e318213d51b.

Liu, X., Tian, X., Wang, F., Ma, Y., Kornmann, M., and Yang, Y. (2014). BRG1 promotes chemoresistance of pancreatic cancer cells through crosstalking with Akt signalling. *Eur J Cancer* 50(13), 2251-2262. doi: 10.1016/j.ejca.2014.05.017.

Liu, X., Zheng, W., Wang, W., Shen, H., Liu, L., Lou, W., et al. (2017). A new panel of pancreatic cancer biomarkers discovered using a mass spectrometry-based pipeline. *Br J Cancer* 117(12), 1846-1854. doi: 10.1038/bjc.2017.365.

Ma, D., Jing, X., Shen, B., Liu, X., Cheng, X., Wang, B., et al. (2016). Leukemia inhibitory factor receptor negatively regulates the metastasis of pancreatic cancer cells in vitro and in vivo. *Oncol Rep* 36(2), 827-836. doi: 10.3892/or.2016.4865.

Maehara, S., Tanaka, S., Shimada, M., Shirabe, K., Saito, Y., Takahashi, K., et al. (2004). Selenoprotein P, as a predictor for evaluating gemcitabine resistance in human pancreatic cancer cells. *Int J Cancer* 112(2), 184-189. doi: 10.1002/ijc.20304.

Miyoshi, E., and Kamada, Y. (2016). Application of glycoscience to the early detection of pancreatic cancer. *Cancer Sci* 107(10), 1357-1362. doi: 10.1111/cas.13011.

Park, J., Choi, Y., Namkung, J., Yi, S.G., Kim, H., Yu, J., et al. (2017a). Diagnostic performance enhancement of pancreatic cancer using proteomic multimarker panel. *Oncotarget* 8(54), 93117-93130. doi: 10.18632/oncotarget.21861.

Park, J., Lee, E., Park, K.J., Park, H.D., Kim, J.W., Woo, H.I., et al. (2017b). Large-scale clinical validation of biomarkers for pancreatic cancer using a mass spectrometry-based proteomics approach. *Oncotarget* 8(26), 42761-42771. doi: 10.18632/oncotarget.17463.

Peng, M., Zhang, Q., Cheng, Y., Fu, S., Yang, H., Guo, X., et al. (2017). Apolipoprotein A-I mimetic peptide 4F suppresses tumor-associated macrophages and pancreatic cancer progression. *Oncotarget* 8(59), 99693-99706. doi: 10.18632/oncotarget.21157.

Rebours, V., Le Faouder, J., Laouirem, S., Mebarki, M., Albuquerque, M., Camadro, J.M., et al. (2014). In situ proteomic analysis by MALDI imaging identifies ubiquitin and thymosin-beta4 as markers of malignant intraductal pancreatic mucinous neoplasms. *Pancreatology* 14(2), 117-124. doi: 10.1016/j.pan.2013.12.001.

Resovi, A., Bani, M.R., Porcu, L., Anastasia, A., Minoli, L., Allavena, P., et al. (2018). Soluble stroma-related biomarkers of pancreatic cancer. *EMBO Mol Med* 10(8). doi: 10.15252/emmm.201708741.

Roberts, A.S., Campa, M.J., Gottlin, E.B., Jiang, C., Owzar, K., Kindler, H.L., et al. (2012). Identification of potential prognostic biomarkers in patients with untreated, advanced pancreatic cancer from a phase 3 trial (Cancer and Leukemia Group B 80303). *Cancer* 118(2), 571-578. doi: 10.1002/cncr.26270.

Shi, Y., Gao, W., Lytle, N.K., Huang, P., Yuan, X., Dann, A.M., et al. (2019). Targeting LIF-mediated paracrine interaction for pancreatic cancer therapy and monitoring. *Nature* 569(7754), 131-135. doi: 10.1038/s41586-019-1130-6.

Sliker, B.H., Goetz, B.T., Peters, H.L., Poelaert, B.J., Borgstahl, G.E.O., and Solheim, J.C. (2019). Beta 2-microglobulin regulates amyloid precursor-like protein 2 expression and the migration of pancreatic cancer cells. *Cancer Biol Ther* 20(6), 931-940. doi: 10.1080/15384047.2019.1580414.

Takano, S., Yoshitomi, H., Togawa, A., Sogawa, K., Shida, T., Kimura, F., et al. (2008). Apolipoprotein C-1 maintains cell survival by preventing from apoptosis in pancreatic cancer cells. *Oncogene* 27(20), 2810-2822. doi: 10.1038/sj.onc.1210951.

Tsuda, M., Fukuda, A., Roy, N., Hiramatsu, Y., Leonhardt, L., Kakiuchi, N., et al. (2018). The BRG1/SOX9 axis is critical for acinar cell-derived pancreatic tumorigenesis. *J Clin Invest* 128(8), 3475-3489. doi: 10.1172/JCI94287.

Xue, A., Chang, J.W., Chung, L., Samra, J., Hugh, T., Gill, A., et al. (2012). Serum apolipoprotein C-II is prognostic for survival after pancreatic resection for adenocarcinoma. *Br J Cancer* 107(11), 1883-1891. doi: 10.1038/bjc.2012.458.

Xue, A., Scarlett, C.J., Chung, L., Butturini, G., Scarpa, A., Gandy, R., et al. (2010). Discovery of serum biomarkers for pancreatic adenocarcinoma using proteomic analysis. *Br J Cancer* 103(3), 391-400. doi: 10.1038/sj.bjc.6605764.

Yu, S., Li, Y., Liao, Z., Wang, Z., Wang, Z., Li, Y., et al. (2019). Plasma extracellular vesicle long RNA profiling identifies a diagnostic signature for the detection of pancreatic ductal adenocarcinoma. *Gut*. doi: 10.1136/gutjnl-2019-318860.

Zeng, H., Yu, H., Lu, L., Jain, D., Kidd, M.S., Saif, M.W., et al. (2011). Genetic effects and modifiers of radiotherapy and chemotherapy on survival in pancreatic cancer. *Pancreas* 40(5), 657-663. doi: 10.1097/MPA.0b013e31821268d1.

Zhang, H., Lv, L., Liu, H., Cui, L., Chen, G., Bi, P., et al. (2009). Profiling the potential biomarkers for cell differentiation of pancreatic cancer using iTRAQ and 2-D LC-MS/MS. *Proteomics Clin Appl* 3(7), 862-871. doi: 10.1002/prca.200800029.

Zhao, J., Patwa, T.H., Qiu, W., Shedden, K., Hinderer, R., Misek, D.E., et al. (2007). Glycoprotein microarrays with multi-lectin detection: unique lectin binding patterns as a tool for classifying normal, chronic pancreatitis and pancreatic cancer sera. *J Proteome Res* 6(5), 1864-1874. doi: 10.1021/pr070062p.

Zhao, J., Simeone, D.M., Heidt, D., Anderson, M.A., and Lubman, D.M. (2006). Comparative serum glycoproteomics using lectin selected sialic acid glycoproteins with mass spectrometric analysis: application to pancreatic cancer serum. *J Proteome Res* 5(7), 1792-1802. doi: 10.1021/pr060034r.
